# Environmental valuation and knowledge production in Swedish marine and water management

**DOI:** 10.1007/s40656-025-00698-y

**Published:** 2025-10-09

**Authors:** Maria Paulsson, Christopher Kullenberg, Lena Eriksson

**Affiliations:** Department of Philosophy, Linguistics and Theory of Science, University of Gothenburg, Gothenburg, Sweden

**Keywords:** Environmental valuation, Knowledge production, Marine management, Valuation practices, Ecosystem approach

## Abstract

This article explores how environmental valuation and knowledge production shape Swedish marine and water management through the case of the Swedish Agency for Marine and Water Management (SwAM). Tasked with conserving, restoring, and sustainably using aquatic resources following an Ecosystem Approach (EA), SwAM navigates complex interactions between these processes and the generation of actionable knowledge. Drawing on perspectives from Science and Technology Studies (STS) and Environmental Ethics, the article explores how SwAM constructs, translates, and operationalizes values in its management strategy and associated action plans, with empirical focus on three cases: seals as obstacles in professional fisheries, the socio-economic value of recreational fishing and tourism, and the prospective value of aquaculture. The analysis shows that SwAM’s interpretation of the EA renders ecological value inseparable from economic benefit, as ecological functions are translated into measurable, often monetary indicators in the pursuit of ‘balance’. Scientific knowledge becomes a prerequisite for valuation but is shaped by a governance logic that prioritizes quantification, standardization, and economic utility. Rather than enabling a plurality of environmental values, this logic tends to privilege those that can be expressed in instrumental and monetizable terms. This raises critical questions about whether the notion of ecological balance, central to the EA, can be realized within a framework that equates environmental worth with economic outcomes. A shift toward non-anthropocentric governance would require not only rethinking valuation practices but also developing epistemologies capable of recognizing non-instrumental dimensions of nature’s value.

## Introduction

At the intersection of science, policy, and society, various actors engage with questions concerning knowledge production and environmental management, particularly regarding how humans relate to nature. In recent years, management of marine resources has been high on the political and scientific agendas, especially in terms of the amount and type of knowledge required to enable their sustainable use. Within this context, familiar questions are once again raised about the boundaries between humans and nature, the production of knowledge centered on human interests, and the framing of nature as a resource for human use.

In response to growing threats to ecosystem viability, the need for new regulatory instruments has been placed on the political agenda. The United Nations Convention on Biological Diversity (CBD), established in 1993, aims to preserve biological diversity, promote sustainable use of natural resources, and ensure the equitable distribution of genetic resources (CBD Secretariat, 2025). To support these aims, the Ecosystem Approach (EA) was proposed as a strategic management framework. It is presented not only as a method for implementing the Convention but as a guiding principle that permeates its goals and strategies (CBD Secretariat, 2004). Central to the EA is a holistic view of ecosystems, emphasizing the interdependence of their components rather than focusing on isolated parts. The approach is operationalized through twelve principles known as the ‘Malawi Principles’, which have shaped subsequent understandings of what constitutes effective marine, water, and fisheries management (CBD Secretariat, 2025).

Within Swedish marine management, the EA has been translated into national guidelines to adapt its principles to local conditions (Swedish Environmental Protection Agency, 2007). Responsibility for marine resources was previously divided between two state agencies with distinct mandates: the Swedish Board of Fisheries, focused on ecologically and economically sustainable fisheries with a strong emphasis on resource use and industry interests (Swedish Parliament, 1996), and the Swedish Environmental Protection Agency, which promoted sustainable development from a broader environmental and conservation-oriented perspective (Swedish Parliament, 2012). While the former prioritized the management and utilization of fish stocks, the latter focused on ecological protection. In 2011, these fragmented responsibilities were consolidated into the newly formed Swedish Agency for Marine and Water Management (SwAM), which was tasked with coordinating, regulating, and monitoring marine management in line with the EA (SOU, 2010, p. 8).

While SwAM is responsible for overseeing the conservation, restoration, and sustainable use of Sweden’s lakes, rivers, seas, and fish resources (SwAM, 2021a), its mandate also requires a comprehensive perspective on marine and water issues, including knowledge generation, conflict management, and the balancing of competing interests (SOU, 2010, p. 8; SwAM, 2021a). Guided by the EA, the agency is required to consider multiple interests and understandings of marine issues, resulting in a need to form new ways of ordering the many values that marine ecosystems are made to represent.

This complex mission makes SwAM a particularly relevant case for studying the implementation of the EA in a national context. It also offers insight into how knowledge is developed, shaped, and applied in a domain marked by diverse and potentially conflicting values. While several actors are involved in implementing the EA, SwAM has an explicit governmental mandate to work with ecosystem-based management, specifically of the marine and aquatic environment. This positions the agency as the principal authority responsible for operationalizing the approach through policy and practice. As part of this role, SwAM is tasked with identifying and valuing marine ecosystem services, making it a key actor in shaping not only how marine resources are governed, but also how their meaning and value are constructed as a resource within an EA framework. This raises the question of what constitutes a “value” in the context of ecosystem-based management, and how such values are formed, negotiated, and made actionable within institutional frameworks.

### Values and valuation

Before examining how values are mobilized in SwAM’s work, we turn to theoretical perspectives that help outline the approach taken in this article. While the definition of value remains contested, we understand it not as something fixed or objective, but as context-dependent and constructed. Following Smith (1988) in *Contingencies of Value*, value is defined as lacking intrinsic, universal, or transcendental qualities. Instead, it emerges from dynamic systems of needs, interests, and purposes associated with the entity in question, systems that are themselves unstable and relational. In this view, the classification of an entity and its function co-constitute one another in a process of mutual transformation.

Smith also revisits the distinction between use value (or utility) and intrinsic value. Use value is commonly linked to efficiency, function, instrumental application, and material benefit, typically grounded in utilitarian logic. Intrinsic value, by contrast, emphasises the worth of an entity regardless of its usefulness. Yet this binary may be misleading if utility is understood as inescapable, when entities considered non-instrumental or unique may ultimately fulfil human purposes, albeit in less direct or measurable ways. Descriptions of intrinsic value still rely on sensory experiences and attributed properties, which may themselves carry forms of utility. The distinction, then, is not simply between instrumental and intrinsic, but between value as a static property and value as something emergent, shaped through interaction and situated practices. These practices may appear stable, yet remain open to reinterpretation and negotiation, including in how value itself is defined.

In valuation literature, the process of assigning value is widely recognized as complex. This complexity arises not only from technical challenges related to measurement (Bingham et al., 1995; Primmer & Furman, 2012), but also from critical perspectives on the reduction of environmental entities to economic terms. Toman (1998), for instance, argues that attempts to quantify ecosystem services often lack adequate grounding in material reality, making it difficult to assess their actual worth. Such critiques suggest that valuation may offer a shaky foundation for policymaking, particularly amid persistent disagreements about what to measure and how. These concerns raise a broader question: is valuation, in its conventional form, even a viable or meaningful endeavor?

Theoretical contributions from pragmatic sociology further complicate this picture by promoting a shift from fixed ‘values’ to ‘modes of valuation’, linking these to different modes of engagement with the environment (Centemeri, 2015). Continuing in line with this approach, environmental valuation can be understood as creating an ‘ecology of values’ composed of diverse ‘appreciations of nature’ (Efstathiou & Myskja, 2019). This notion refers not only to instrumental or non-instrumental attachments, but also to complex entanglements that emerge from the issues at stake.

According to Isacs et al. (2023), examining how values are created and attributed is essential for understanding choices in environmental management, as value pluralism can both inform and complicate decision-making. Valuation practices thus offer a lens into the understandings that underlie policy, especially in contexts marked by competing interests. These practices are thereby social practices, which are participating in and contributing to the ordering of society and could, according to Helgesson and Muniesa (2013), be studied as such.

This article does not seek to determine whether valuation is desirable; rather, we examine how and why valuation occurs in various forms. We apply a ‘valuographic’ analysis, a theoretical and methodological framework that investigates how values are made actionable and explores their practical implications. Our approach adopts a pragmatist stance, focusing on the act of valuation and on how values are enacted in practice (Dussauge et al., 2015a, 2015b).

Valuation practices are closely intertwined with defining and representing problems (Dussauge et al., 2015; Bacchi, 2012a). While problems are often presented as fixed, self-evident, and technical, they are inherently political constructs that shape how we perceive and approach the world (Bacchi, 2012a, 2012b). Policy documents and strategies become tools that shape and govern innovation within organizations and society (Asdal, 2015a, 2015b). They thus offer an entry point for analyzing how institutions define problems and propose solutions, both of which play an active role in producing the realities they claim to address.

When studying marine management of a governmental agency situated at the intersection of science, policy, and society, practices that may appear purely epistemic can also be understood as valuation practices. For example, developing measurable ecological indicators to assess marine environmental status not only shapes what counts as scientifically valid knowledge, but also influences how marine features are valued and managed (Turnhout et al., 2007). Different epistemic communities are shown to influence valuation through competing value ontologies (Craig et al., 2019). How values are made is clearly intertwined with what knowledge will be recognized as valuable, making valuation an inseparable part of knowledge production (Dussauge et al., 2015a, 2015b; Polk & Knutsson, 2008).

In the following section, we analyze how SwAM constructs, prioritizes, and operationalizes values in the management of aquatic resources. We focus on how valuation unfolds within one of SwAM’s key policy documents: *The Strategy for Swedish Fisheries and Aquaculture 2021–2026 – Healthy Ecosystems and Sustainable Use* (hereafter “the strategy”). Developed in collaboration with the Swedish Board of Agriculture, the strategy responds to a governmental mandate to coordinate stakeholder efforts in marine and aquatic governance (SwAM, 2021b). While the EA, as articulated in foundational frameworks like the CBD, aspires to balance instrumental and intrinsic values, this study investigates how that balance is realized and put into practice through SwAM’s strategy. Our focus is on how SwAM attributes value to resources, and how its implementation of the EA enacts the broader aim of maintaining ecological and societal balance.

## Valuing marine ecosystems

Since the withdrawal of the ice sheet about 13,000 years ago, the Swedish coastline has been utilized by human populations for subsistence. However, it was not until the early modern period that fishing began to be systematically regulated. This shift occurred as the Swedish monarchy increasingly recognized fisheries as a source of national economic value. During the 18th century, concerns arose regarding the country’s trade balance in fish products, as imports significantly exceeded exports, which was viewed as a threat to economic stability. In response, the state began investing in scientific studies aimed at enhancing the efficiency of fisheries. These early investigations not only sought technical improvements but also speculated on the ecological consequences of overfishing, marking an early awareness of sustainability challenges (Ask & Svedäng, 2019).

Building on this regulatory concern, the role of technology and state governance became increasingly prominent throughout the 19th and 20th centuries. From the late 19th century until the 1990s, fishing efficiency increased dramatically. At the beginning of this century, industrialization contributed to developing new fishing methods and establishing authorities to manage the fisheries. During wartime, especially, there was a significant decrease in fishing activity and a substantial increase in fish stocks, which was interpreted as support for the anticipated long-term effects of overfishing. Following that period, a major technological push, driven by growing interests from the fishing industry, and a fishing policy aimed at meeting domestic fish needs and promoting exports, led to a significant upscaling of professional fisheries. Technical innovations were often met with great enthusiasm and financial support from the Swedish government because they were considered to benefit essential societal concerns.

These developments in Sweden’s fishing industry, marked by accelerated exploitation and technological advancement, highlight the growing tension between economic growth and ecological sustainability. Meanwhile, current policy frameworks such as the CBD, which aim to manage marine resources in ways that explicitly incorporate both ecological and socio-economic values into management, had already been developed at a global level. Articulated as the EA, the CBD’s preamble states a commitment to both instrumental and intrinsic values of biodiversity, where ‘ecological’ values beyond the instrumental benefits of nature are emphasized as necessary components for a more sustainable valuation (CBD Secretariat, 2004).

Throughout the early 2000s, Sweden began to slowly incorporate the EA, developing institutions and regulations for implementing EU directives and establishing SwAM as a key actor for managing aquatic resources (Österblom et al., 2017). When translated into its management strategy, SwAM makes explicit the purpose of applying the EA for a management that considers environmental, economic, and social sustainability. The EA is thereby posed as a tool for balance, shifting from an exploitative toward a more holistic approach to interacting with nature.

SwAM’s overreaching management strategy results in three action plans: one for professional fisheries, a second for recreational fishing and fishing tourism, and a third for aquaculture. The first two, concerning professional and recreational fishing activities, are structured based on three overarching goal areas: well-managed and functional ecosystems; competitiveness, profitability, and social values; followed by knowledge and communication, while the action plan for aquaculture presents a non-categorized list of its various goal areas. The values outlined in the strategy are exemplified in the action plans through concrete cases that illustrate how these values can, or cannot, be operationalized and made commensurable to achieve balance.

SwAM describe stakeholder involvement as a central part in developing the strategy, contributing to both its content and design through participation in a stakeholder dialogue processes and by providing continuous feedback (SwAM, 2021b, p. 41). In this context, stakeholders include governmental authorities and other relevant actors at the national, regional, and local levels. This encompasses representatives from the fishing and aquaculture sectors, including academic institutions and research institutes, public organizations such as municipalities, county administrative boards, and various governmental agencies, as well as industry associations related to fishing, organizations and businesses within aquaculture, and environmental and nature conservation groups. Given the diversity of organizations involved, it is reasonable to assume that they represent a broad spectrum of values, some of which may overlap. However, the documents do not clearly attribute specific values to each organization, as stakeholders are grouped in different ways within the action plans. Some obvious examples can still be identified, such as the “economic” values associated with commercial fishers (SwAM, 2021c).

In a context dominated by economic metrics, ecological values must be quantified to carry equal weight in policy and management. When values are more easily rendered measurable, they can be compared and balanced against one another, and potential solutions and follow-up actions are articulated. In contrast, values that are difficult to quantify resist such balancing and signal a need for further knowledge to support measurability. As the strategy notes, “[T]here is currently a well-established system for generating and reporting biological data to relevant authorities, such as through ICES and the Swedish University of Agricultural Sciences. However, there is no equivalent system for social and economic data” (SwAM, 2021c, p. 49). Aquatic resources are more readily expressed in economic terms like growth and profitability, but an EA calls for a broader form of balancing. This drives efforts to develop indicators that capture ecological and social dimensions in ways that can shape decision-making.

The ‘balance’ embedded in the EA, as it is applied through SwAM’s strategy, depends on translating diverse values into a shared system of measurement. In doing so, the notion of ‘balance’ shifts: from a negotiation among incommensurable value types to a form of technical equivalence grounded in managerial rationality, where management prioritizes quantifiable outcomes and operational efficiency. To explore how these dynamics unfold in practice, the next section draws on specific action plan formulations that we use as examples to illustrate how marine ecosystem elements are redefined as forms of value through policy. In doing so, we examine how SwAM’s use of the EA shapes a particular understanding of ‘balance,’ one that reflects the difficulties of converting varied value types into actionable management tools.

### Seals as economic obstacles

In SwAM’s strategy document, professional fishing is presented as essential for generating a range of social, cultural, and especially economic values. Rather than highlighting tensions between recreational, tourism-based, and professional fishing, the strategy emphasizes shared challenges, most notably, the deteriorating condition of aquatic ecosystems and declining fish populations. Fishing and aquaculture, despite internal conflicts, are portrayed as interconnected parts of a larger societal ecosystem:

Even though there are conflicting interests within and between the fishing and aquaculture industries, there is also significant potential for collaboration and synergies. For instance, local professional fishing is a prerequisite for maintaining the port infrastructure that recreational fishing and fish tourism depend on. Professional fishing also serves as a carrier of culture and traditions, contributing to Sweden’s appeal to tourism and recreation through vibrant harbors with active fishing and fish products for sale (SwAM, 2021b, p.12).

SwAM’s management framework centers on fostering collaboration across stakeholder groups with varying interests by emphasizing interdependence and shared responsibility for long-term viability. Rather than distinguishing between small-scale and industrial operations, the strategy categorizes actors by function: those who fish professionally, those who fish recreationally, and those involved in marine farming. Within this structure, professional fisheries are portrayed as foundational to coastal communities, supplying food, employment, and cultural continuity:

Swedish professional fishing contributes to a vibrant rural area, coast, and archipelago by providing employment opportunities within fishing, processing industries, and other related sectors. For many coastal communities, local professional fishing is a crucial part of the region’s identity and enhances the attractiveness of municipalities for residents and tourists (SwAM, 2021b, p. 2).

However, their economic sustainability is increasingly threatened by the reduction of marine resources, an issue that also affects recreational fishing and tourism, both reliant on healthy fish stocks. Among the challenges detailed in the action plan for professional fisheries is the growing seal population, which is presented as harmful to fishing by damaging gear and reducing catch. SwAM calls for more targeted and effective seal hunting, while acknowledging the need for further investigation into appropriate responses to the issue:

In spite of the majority in the fishing industry considering seal […] populations a significant concern, the quotas for hunting are not fully utilized. This may be due, in part, to the difficulty and cost of seal and cormorant hunting, as well as the prohibition on selling seal products (SwAM, 2021c, p. 21).

Notably, seals are not assigned a direct economic value, hunting is costly and the sale of seal products remains prohibited, yet they are framed as ecologically disruptive to economically important human activities. The lack of economic incentive is mentioned as a reason why hunting quotas remain underused. In this framing, the seal emerges as an obstacle to profitability, viewed only through its impact on human-centered activities. Largely absent, however, is any articulation of the seal’s potential positive ecological role within the marine system, even less as a bearer of an intrinsic value.

While current policy focuses on mitigating economic damage, SwAM also acknowledges key knowledge gaps, particularly concerning the long-term ecological consequences of culling and the status of both fish and seal populations. These gaps are framed not as ethical or conservationist objections, but as practical uncertainties about ecosystem function. SwAM states that it will “[C]onduct a scientific evaluation of the effects of population-regulating seal hunting from an ecosystem perspective” (SwAM, 2021c, p. 22), signaling the need for scientific evaluation to understand how seals operate within marine ecosystems.

In this framing, the seal becomes a site where ecological complexity is translated into managerial terms. Its role must first be scientifically defined before any action is deemed legitimate, making science the gatekeeper for transforming ecological interactions into policy interventions. Rather than disrupting the dominance of economic priorities, the ‘ecologically valuable seal’ is made actionable only once it fits within a framework of measurable impacts. What emerges, then, is a form of valuation that privileges instrumental rationality, where ecological considerations are acknowledged, but only insofar as they can be aligned with economic logic.

### Profitable salmon and trout

The material and cultural infrastructure surrounding local professional fishers also supports adjacent sectors like recreational fishing and fishing tourism. According to SwAM’s second action plan, these activities contribute to employment, tax revenue, entrepreneurship, nutritious food, attractive environments, and overall public well-being. Declining fish stocks and deteriorating marine ecosystems threaten not only professional fisheries but also these broader social benefits, since “[…] a widespread interest in recreational fishing, along with well-managed fish stocks, is a fundamental prerequisite for regional development through fish tourism, while also contributing to public health and social cohesion” (SwAM, 2021b, p.2).

Reduced profitability affects various nodes in fishing-related commodity chains, undermining the vision of a ‘vibrant rural area’ and challenging the identity and attractiveness of coastal municipalities. Recreational fishing and fishing tourism generate both cultural and social values, which can be translated into monetary terms, such as income from guided tours, accommodations, restaurants, and equipment sales. All these values, however, hinge on the ecological condition of fish stocks, such as salmon and trout, whose biological functionality is instrumental to enabling economic and social benefits:

Thanks to extensive measures involving both biological restoration in our watercourses and restrictions on commercial fishing, our fish stocks have been given the opportunity to recover from very low levels over the past 30 years. However, for the majority of salmon stocks, and also for trout, there is still a long way to go before the goals are achieved. As more salmon and trout return to their rivers and streams, the interest from local and visiting recreational fishermen to take advantage of this resource has increased. Strong and viable stocks create conditions for attractive fishing. Fishing, in turn, makes it more appealing to live along our often sparsely populated river valleys, but also provides opportunities for developed fishing tourism and employment (SwAM, 2021d, p.16).

SwAM emphasizes that a larger number of salmon and trout in Swedish rivers and streams would increase interest from local and visiting recreational fishermen to make use of these resources. This would contribute to making fishing more attractive for the population, encourage people to settle around Swedish waters and utilize such areas, while also creating more job opportunities. SwAM invokes 50–70 million fishing enthusiasts and 10,000 miles of coastline to make the case that fishing tourism is an underdeveloped product of great growth potential. Actualized economic profits for commercial fishing are framed as something of need to be balanced against potential values generated by recreational fishing and fishing tourism.

In this action plan, recreational fishing and fishing tourism are shown to be transformed into economic, social, and cultural values and made measurable in the form of hotel bookings, trade for local businesses and a restored and reinvigorated local way of life in fishing villages along the coast. Here, salmon and trout are framed as ecological enablers of downstream benefits in the form of tourism, local identity, and economic development, dependent on suitable conditions for well-functioning and well-developed fish stocks. Further developing stock-specific management, which implies management tailored to the needs of each stock, is presented as a suitable solution for conserving salmon and trout stocks and stimulating their development.

However, SwAM also highlights a lack of knowledge about the potentially negative effects of these activities, which could occur both in connection with fishing itself and in the utilization of the environment during other leisure activities. Fishing is described as one of Sweden’s most popular leisure activities, and changes might give rise to yet unknown societal effects. Building on that understanding, SwAM calls for more knowledge regarding the socio-economic values and impacts of recreational fishing and fishing tourism, as well as their effects on aquatic ecosystems. Filling such ‘knowledge gaps’ becomes a necessity for subsequently balancing economic, social, and cultural values against ecological values. In this framing, salmon and trout are valued not in themselves, but for their capacity to support other forms of value generation, thus illustrating how ecological values are mobilized instrumentally to promote societal goals.

### Future values

Beyond established but threatened resources, SwAM articulates the need to also create favorable conditions for future values. The development of Swedish aquaculture is described as underperforming relative to its potential to generate social and economic benefits. As noted, “[Aquaculture is] a significant industry with substantial growth potential in the Swedish countryside (SwAM, 2021b, p. 2). Aquaculture is presented as a potential contributor to food production, public health, environmental well-being, and the bioeconomy. Yet, it has not developed as desired, motivating efforts to position it as a future pillar of the ‘blue economy’ (SwAM, 2021b, p. 2).

The underdeveloped state of Swedish aquaculture includes a lack of designated areas for its utilization. These issues have resulted in a need for site planning, something that many municipalities refrain from doing due to resource and knowledge constraints. This has created a demand for more data to identify such areas and more knowledge about the potential positive and negative impacts of aquaculture on ecosystems. Here, as noted in the previous examples, ecosystems are largely framed as instrumental service providers, with their health seen as a necessary condition for realizing aquaculture’s promised returns.

Planning for the development of aquaculture and the potential values it can contribute, thus requires knowledge not only on its productivity compared to other food systems but also on its environmental impacts. While it is positioned as a source of food and economic gain, its sustainability depends on how its effects on ecosystems are understood and managed. However, significant knowledge deficiency is stressed, not just about its impacts, but also about how to assess them. This epistemic uncertainty undermines the ability to weigh ecological risks against projected economic benefits, calling for the need for more robust evaluation methods such as improved environmental indicators and more refined tools for ecological assessment.

Balancing aquaculture’s potential economic values with its ecological footprint depends on the development of quantifiable metrics. As with salmon, trout, and seals, ecosystems are viewed through an instrumental lens where their value is derived from the services they enable, rather than from some kind of intrinsic value. In this framing, the future value of aquaculture hinges on the ability to operationalize both its contributions and its costs within a broader valuation framework rooted in an economically driven managerial rationality.

## Balancing values

Having outlined SwAM’s implementation of the EA in its attempt to balance diverse values attributed to marine environments, the following sections examine how this notion of ‘balance’ is constructed and operationalized. The analysis unfolds in three parts: first, how ecological value is framed instrumentally and tied to economic outcomes; second, how measurability becomes central to enabling valuation and decision-making; and third, how these logics together produce an economically grounded ecology, where nature’s worth is largely defined by its contribution to societal and economic goals.

### Redefining balance

Within environmental policy, values such as ‘ecological’, ‘economic’, and ‘social’ are often treated as analytically distinct, yet their meanings overlap and shift depending on context. Ecological values, for instance, may be framed in both intrinsic terms, as ends in themselves, and instrumental terms, as supporting human well-being. As articulated by the CBD, the EA’s balancing function requires weighing ecology’s utility to humans against its intrinsic worth. The first of the twelve principles on which the EA was founded emphasizes that “[E]cosystems should be managed for their intrinsic values and for the tangible or intangible benefits for humans, in a fair and equitable way” (CBD Secretariat, 2004, p. 8). This involves framing marine environments simultaneously as entities with intrinsic value and as resources that, when optimized for economic, social, and cultural benefits, provide services to humans.

Furthermore, the tenth principle describes a kind of balance between conservation and the use of biodiversity, portraying biodiversity as both something that maintains ecosystems, as an intrinsically valuable resource and as a fundamental necessity for all life. This balance explicitly involves weighing the benefits we may attribute to marine ecosystems against the conservation values that can be expressed in terms of a more purely ‘ecological’ value compared to an economically oriented valuation of ecology.

In the CBD, ecosystems are also assigned a form of utility value, which is linked to the concept of ‘ecosystem services’. Under the approach’s fifth principle, ecosystem services are understood as an expression of the relationships within and between species, between species and their abiotic environment, and to human well-being. Additionally, ecosystem services are tied to the multitude of benefits that emerge from these dynamic interactions, benefits that fundamentally depend on biodiversity and long-term conservation. In this approach, marine environments encompass not only a plurality of values as resource for both human and non-human benefits, but also as bearers of intrinsic value.

In its applied form, the EA as practiced by SwAM attributes primarily instrumental, monetary values to nature, framing ecosystem services as benefits for humans. This perspective is evident in examples such as professional fishing, recreational fishing, fish tourism, and aquaculture, where healthy marine ecosystems are frequently portrayed as foundational for economic development, job creation, and population retention in coastal areas. Such framing underscores the societal importance of marine environments but also illustrates how ecological value is often understood through its role in enabling human-centered outcomes. Building on this logic, SwAM emphasizes the economic, social, and cultural values that marine environments can generate when treated as resources to be optimized.

When the sea’s degraded state threatens fish stocks, management guided by the EA is presented as a solution, one that requires integrating ecological, economic, and social dimensions of sustainability. By assigning different types of value to marine life, SwAM creates a framework for weighing competing interests. These trade-offs then form the basis for developing policy measures that address both environmental concerns and the utilization of marine resources.

Central to this balancing act are strong fish stocks, which not only generate economic value through commercial fisheries, but also support the social and cultural benefits associated with recreational fishing and tourism. Within this framework, the notion of ‘balance’ emerges as a guiding principle for navigating tensions between ecological sustainability and human use.

Yet this balance increasingly revolves around instrumental values converted into profit. What becomes apparent is a shift: the original idea of balance embedded in the EA and SwAM’s management mandate is transformed. As ecological value becomes closely tied to, if not indistinguishable from, economic benefit, it loses its footing as a distinct category. Rather than balancing ecological and economic values, SwAM’s logic centers on negotiating between different kinds of human-centered benefits, some of which are dependent on healthy ecosystems. In this process, ecology is tightly interwoven with economy, where the integration of values intended by the EA results in ecological worth becoming nearly unrecognizable as something intrinsic or separate from monetary outcomes.

### Translation through measurability

A central pillar of the EA is scientific knowledge production. As part of its defining principles, it is stated that the approach should be based on applied scientific methods aimed at capturing the structures, processes, and functions occurring in the interactions between organisms and their environment. Within SwAM’s application of the EA, a recurring theme is the transformation of ecological knowledge into something legible, comparable, and actionable, primarily through measurability. Scientific knowledge plays a central role, not only as a foundational element of the EA, but also as a means of shaping ecology into a form that can be aligned with economic and social goals. Knowledge, in this context, functions as content moulded to fit a predefined framework that prioritizes management and governance.

The EA is described as a science-based approach, rooted in applied methods that seek to understand the dynamic interactions among organisms, including humans, within ecosystems. This inclusive framing recognizes humans as integral components of ecological systems. However, in the policy documents, knowledge appears less as an open-ended pursuit and more as an instrument for generating data that supports balancing competing values. This becomes particularly apparent when ecological, economic, and social values are weighed against each other. Although the EA aspires to accommodate multiple forms of value, implementation tends to emphasize those that are most easily quantified. In practice, this often results in a privileging of economic measures, such as financial profitability or job creation, over less tangible or less measurable ecological values.

For SwAM to achieve ‘balance’ within this framework, values must be made measurable. The logic is clear: without standardized indicators or quantifiable metrics, trade-offs cannot be calculated, and policy decisions cannot be justified. Thus, measurability becomes the condition for valuation. In the case of aquaculture, for instance, the lack of certainty about how to quantify certain social or ecological impacts complicates the effort to weigh risks and benefits. This uncertainty underscores the broader dilemma of how to operationalize ecological knowledge when not all values can be clearly measured or translated into economic terms. By linking value to quantifiable outputs, like revenue, employment, or population retention, marine ecosystems are increasingly framed as resources for human use. In this framing, ecology functions less as a domain with intrinsic worth and more as an enabling platform for economic and social benefit. Even ‘ecological’ value, in this context, becomes instrumental: important not for its own sake, but for its contribution to measurable outcomes.

Furthermore, SwAM’s documents tend to also emphasize the need for “more knowledge” to inform decision-making. But in practice, this often means generating more measurable knowledge. Standardized indicators become tools for translating the complexity of marine ecosystems into categories that can be ranked, compared, and optimized. As a result, those values that are less amenable to quantification, such as the intrinsic worth of biodiversity or the ethical imperative to protect nature, risk being marginalized or overlooked entirely.

Frameworks like ecosystem services attempt to bridge this gap by converting diverse ecological and social functions into monetary equivalents. While this enables integration into economic policy and planning, it also risks flattening value pluralism into a single dimension, where only what can be priced counts. The result is a tension at the heart of the EA: between the promise of inclusive, knowledge-based management and the practical dominance of measurable, instrumental outcomes. In this way, ecosystems are rendered into utilities, for human benefit above all. The challenge that emerges is whether, in striving to balance values, the EA ultimately transforms the very meaning of ecological value into something inseparable from economic rationality.

### An economically grounded ecology

Through SwAM’s policy documents, it becomes evident that marine resources are primarily framed in terms of the benefits they provide to humans, above all, economic ones. This framing constitutes a form of instrumental valuation, where marine environments are treated as resources that generate services and utilities convertible into economic assets. They are thus assigned monetary aspects such as growth, profitability, competitive food supply chains, production development, job creation, and efficiency. These priorities align closely with the broader interests of ‘industry’, which SwAM’s texts explicitly reference through goals such as sustainable solutions, innovation, and increased knowledge, all cast in relation to economic development.

This logic of value, what might be called a ‘utility logic’, also extends to how ecological dimensions are presented. Marine environments are attributed ecological value, but this value is primarily defined through terms like sustainable use, balanced ecosystems, and ecosystem resilience, all of which are tied to the generation of societal benefit. Ecological value, in this sense, is rarely articulated outside its usefulness to society, most often in economic terms. In contrast, references to ecological value as understood in the foundational principles of the EA, values not directly tied to human use but grounded in intrinsicality, are largely absent.

Describing marine environments as a resource, say even an (ecosystem) *service*, reflects the diverse interpretations of the values attributed to them. Typologies are developed to classify these values, all of which are considered necessary for achieving balance in a societal ecosystem that includes priorities such as growth, employment, health, cultural heritage and tradition. This framing aligns with what Asdal and Huse (2023) describe as a ‘nature-made economy’, where nature actively shapes economic possibilities and constraints rather than serving merely as a passive backdrop. Translating ecological functions into quantifiable terms thus becomes essential for integrating them alongside economic and social metrics.

When the type of ecological value being balanced is largely instrumental. Ecosystems and marine organisms are depicted in terms of the benefits they provide to humans, whether as services, extractable resources, or stabilizing forces within society. In this way, ecological value is tightly aligned with economic and social utility. Marine species and ecosystems are rendered valuable because of their function within human systems, rather than for any inherent or non-utilitarian reason. Intrinsic values, those grounded in the idea that marine life has worth independent of its benefit to humans, are either underrepresented or absent. Where ecology does appear, it often serves as a justification for achieving human-centered outcomes.

This framing has significant implications for how the principle of ‘balance’ is understood within the EA. Rather than representing an ethical equilibrium between diverse and sometimes incommensurable forms of value, balance becomes a managerial process, a strategic weighing of competing human interests. Ecological references, while present, are subordinated to this human-centric logic, reinforcing a valuation regime in which nature’s role is to support societal needs and ambitions.

This economically grounded ecology is thus perpetually dependent on what occurs in and around the marine environment. If one societal actor, for example, exceeds the Maximum Sustainable Yield (MSY), disrupting the reproductive capacity of certain species, that ecological imbalance is mirrored in the societal ecosystem. SwAM’s policy documents suggest that the relationship is reciprocal: if all parts of the societal ecosystem are adequately nourished by marine resources, the entire system will thrive, even grow, following the underlying economic imperative. This conceptualization of marine life and environments as instruments for human ends raises critical questions about the nature of value in the EA. These questions, particularly the tension between anthropocentric and less or non-anthropocentric forms of valuation, will be further explored in the following section.

## Valuation in and beyond the anthropocene

Decision-making in response to the ecological crisis presents the challenge of integrating ecosystems into socio-economic processes (Mouysset, 2023). In marine management, particularly under the EA as defined by the CBD, tensions emerge. On the one hand, certain actors must be enrolled around the shared goal of sustainable resource use; on the other hand, human interests tend to dominate interactions with nature. These tensions reflect longstanding debates on the relationship between society and nature, humans and non-humans, in various disciplines, not least in Science and Technology Studies (STS) and Environmental Ethics (Collins & Yearley, 1992; Callon & Latour, 1992; Singer, 1993; Taylor, 2008). To deepen the analysis of SwAM’s valuation processes, we combine insights from Environmental Ethics to examine the ethical premises of the EA and its divergence from SwAM’s implementation, and from STS to explore how these differences influence knowledge production and environmental decision-making.

To understand how ethical framings influence environmental decision-making, STS offers useful tools, particularly through Bruno Latour’s Actor-Network Theory (ANT), which provides a relational ontology where human and non-human actors, such as animals, ecosystems, technologies, and institutions, are active participants in knowledge production (Latour, 2007). Latour’s flat ontology dissolves artificial human-nature and natural-social dichotomies, highlighting complex interactions within networks. Here, knowledge emerges through processes of translation, where actors shape and reshape one another. In contrast, anthropocentric knowledge production situates humans as central actors, treating nature as a passive resource to be observed, measured, and utilized, often via frameworks like ‘ecosystem services’. A Latourian, non-anthropocentric approach would, instead, recognize nature as an active participant in decision-making rather than a mere resource for exploitation.

These conceptual debates are not confined to theory but also surface in applied contexts, such as marine and environmental sciences, where the challenge of understanding and managing complex ecosystems becomes highly tangible. In these fields, the difficulties in fully grasping marine ecosystems and their significance to human societies are often prominent. Although underwater life is frequently portrayed as mysterious and elusive, it is simultaneously regarded as essential to human well-being. Consequently, efforts are made to assign value to these ecosystems, a process that becomes particularly fraught when navigating the intricacies of human-nature relations. One approach to mediating this is the already mentioned ecosystem services framework, which seeks to capture and categorize the diverse benefits ecosystems provide to human societies by translating ecological functions into human-centric value metrics (Costanza et al., 2017; Gee, 2019; Rozwadowski, 2005; Paramio et al., 2015).

This translation process can result in the commodification of the ocean, where marine life is increasingly understood in terms of market value. Gómez-Baggethun et al. (2010) trace this development historically, showing how ‘ecosystem services’ evolved from early conservation concepts into frameworks for market-based instruments and payment schemes. This commodification raises questions about which aspects of nature are valued, and which are excluded, when economic logic dominates.

The field of Environmental ethics encompasses both anthropocentric and non-anthropocentric perspectives. An anthropocentric, consequentialist view positions humans as managers with superior moral status over nature (Ariensen, 1993). A key distinction in this field concerns intrinsic values or non-instrumental values, which exist independently of human utility. Using the taxonomy of Arias-Arévalo et al. (2018), the valuation of marine ecosystems as an ecosystem service reflects different ways of relating to nature. In SwAM’s strategy and action plans, ‘ecosystem services’ are primarily framed through instrumental values aligned with a ‘gaining from nature’ perspective, such as the creation of seals as economic obstacles. A ‘living in nature’ perspective also appears, incorporating relational values like cultural heritage, identity, and ecological resilience, for example, in references to the profitable salmon and trout. However, a ‘living for nature’ approach, which emphasizes nature’s rights and intrinsic values, is largely absent in SwAM’s valuation frameworks.

Research on knowledge gaps in the valuation of marine ecosystems, particularly from post-anthropocentric perspectives on fish stocks, raises important questions about how value can be understood beyond the framework of ‘ecosystem services’ (Obregón et al., 2018). Within Environmental ethics, perspectives that seek to de-center human interests emphasize ecological sustainability as a value in itself. Deep Ecology, for instance, promotes a biocentric ethic, arguing that all living beings possess an equal right to exist (Næss, 1973), thereby challenging the anthropocentric foundations of the EA and advocating for governance models that recognize nature’s intrinsic worth rather than its instrumental utility.

The ‘Rights of Nature’ (RON) is another approach that exemplifies this by offering a legal and biocultural framework that resists commodification, recognizing ecosystems for their inherent vitality (Nash, 1989). These perspectives collectively challenge dominant sustainability discourses that equate environmental value with economic growth, highlighting the risk of reducing nature to a means to human ends when its worth is defined primarily through utility. In contrast, more ecologically centered approaches call for the prioritization of long-term sustainability and the meaningful integration of local knowledge, elements that were foundational to the original conception of the EA under the CBD, but which remain underutilized in practice.

This shift reorients the focus toward nature’s rights, redefining problem representations and goals. A less anthropocentric approach would emphasize preservation and restoration, grounded in a moral argument for protecting nature rather than an economic rationale for exploitation or resource optimization. However, intrinsic values imply a fixed, inherent worth in nature, independent of context, something that may conflict with Latour’s relational ontology and constructivist epistemology. It is therefore important to distinguish how something is assigned value (its process of valuation) from what kind of value it represents (e.g., intrinsic, instrumental, or relational). This distinction sheds light on the normative assumptions behind environmental decision-making and highlights the importance of understanding valuation not as a static property, but as a dynamic and context-dependent process.

As Craig et al. (2019) argue, economic-utilitarian valuation prioritizes market-based assessments, while value-pluralist approaches recognize incommensurable forms of worth, such as cultural and intrinsic values. Policy reliance on economic valuation often stems from strategic aims to gain political traction, as is evident in SwAM’s framing of marine resource trade-offs. Through this framing risks oversimplifying complex socio-ecological relationships, a challenge already acknowledged within the EA’s ambition to incorporate non-economic values, it highlights a core dilemma in implementation: while economic valuation may facilitate policy action, it risks narrowing the scope of what is considered valuable, thereby undermining the EA’s commitment to holistic and inclusive ecosystem management.

A useful way to study knowledge production in marine management is to examine how valuation frameworks shape human-nature relationships. Arias-Arévalo et al. (2017) argue that “intrinsic, instrumental, and relational values coexist in people’s narratives about the importance of ecosystems.” This paper builds on that argument by suggesting that such narratives have world-ordering effects. The shift in STS toward post-anthropocentric ontologies, epistemologies, and methodologies offers new imaginative possibilities for rethinking marine management. When marine ecosystems are primarily valued in economic terms, as in SwAM’s case, an anthropocentric ontology is enacted. This reinforces the need, as Kopnina (2014) argues, for alternative ontologies that can recognize the intrinsic value of non-human life.

The EA encourages a balance between intrinsic and instrumental values (CBD Secretariat, 2004), yet SwAM’s implementation remains ethically rooted in anthropocentrism, where nature’s value is tied to human needs. While SwAM prioritizes economic values, the question remains: what would a less anthropocentric governance model look like? Such a model would require understanding ecosystems as networks of interacting actors rather than isolated entities (Latour, 2007; Haraway, 2016). For example, while a fish may be viewed as food for humans, it is also food for seals, indicating its embeddedness in a wider ecological web. This functional role does not equate to instrumental value but reflects causal interdependencies within ecosystems that contribute to systemic resilience.

As an actor, SwAM must translate and coordinate diverse interests. The concept of “stake-making” illustrates how assigning economic value to nature also establishes stakes (Dussauge et al., 2015a, 2015b). Valuation of ecosystem services exemplifies this process by framing nature’s worth through its benefits to humans. Fish stocks, for instance, can be tied to direct market value through consumption, whereas their cultural or recreational value is more difficult to monetize. This complexity highlights how valuation often involves extended “chains of effects from ecosystem services to human welfare” (Costanza et al., 1997, p. 255).

Although the EA aspires to integrate multiple forms of value, it is frequently criticized for privileging economic over ecological and ethical dimensions. Some argue that moral imperatives, such as the belief that no one should go hungry, may necessitate valuing nature in economic or social terms (Costanza et al., 1997). Similar arguments arise in marine management, supporting the attribution of instrumental value to nature. These perspectives point to the importance of recognizing nature’s role in supporting human societies, while also promoting coexistence over exploitation. Alternatives to growth-centered valuation, such as the concept of degrowth, challenge the notion that economic expansion must guide environmental governance (Arias Schreiber et al., 2020). Integrating such perspectives may help shift priorities toward long-term ecological resilience, justice, and sustainable coexistence.

## Concluding remarks

With the establishment of SwAM, different mandates and management logics have been unified under the task of sustainably managing Sweden’s marine, water, and fisheries resources in accordance with the EA. Positioned within a complex landscape where a multitude of interests must be coordinated, SwAM seeks to produce knowledge that, in turn, links balanceable values to marine ecosystems. As predefined by the CBD, the approach aims to balance a plurality of values to ensure the preservation of ecological balance. However, when the EA shapes and is shaped by the agency’s management strategy, the challenges of maintaining the idea of balance, where ecology holds equal weight with economy, become evident. This balance unfolds entirely within an anthropocentric framework, where what is termed ecological, economic, and social values are all interpreted as human benefit and profit. Rather than incorporating fundamentally different ethical commitments, such as intrinsic versus instrumental value, SwAM’s implementation balances various forms of utility for humans.

Perhaps the very idea of humans as managers of nature is incompatible with the kind of balance the EA, as predefined by the CBD, advocates. This suggests that the very premise of pluralistic valuation is undermined, as intrinsic values are translated into instrumental logics to fit within a management apparatus grounded in economic rationality. Alternatively, balance in this context might refer to a different form of reciprocal exchange: maximizing the benefits ecosystems provide to humans while minimizing human impact on ecosystems.

Knowledge itself becomes a prerequisite for valuing marine ecosystems, both within the EA by the CBD and in SwAM’s application of it through its policy documents. In this case, scientific knowledge translates into a focus on measurability, where values must be quantifiable to be actionable in policy. This prioritizes economic and easily measured outcomes, marginalizing ecological values that resist standardization. As a result, ecosystems are increasingly framed in instrumental terms, as resources for human benefit, raising critical questions about whether the approach ultimately reshapes ecological value to fit economic rationality.

This article also shows that valuation and epistemic practices are mutually constitutive: valuation requires knowledge, and knowledge is shaped through valuation. For instance, understanding how many seals there are, what they eat, and how that impacts fish stocks is essential for assigning value in policy contexts. Yet the very act of deciding what knowledge is needed, and about whom, is guided by prior valuations. When fish are made valuable through management goals, seals become a knowledge object in relation to that value. This framing not only influences what is being valued but also how knowledge about ecosystems is constructed, prioritized, and mobilized. Valuation practices thus operate as epistemic practices, involving representations that highlight certain aspects while obscuring others. In this case, they function as instruments of anthropocentric governance, translating complex ecological relationships into human-centered benefits. What becomes obscured in this process are alternative ontologies and ethical commitments, such as those grounded in biocentrism or reciprocity, that could challenge the prevailing economic-utilitarian framing.

Along with several of the twelve principles underlying the EA emphasizing the importance of including those most directly affected by management measures in the decision-making processes, SwAM also attempts to integrate local knowledge and interests into its management strategy. The idea behind this is that such decentralization will promote efficiency, effectiveness, and equity, as locally anchored management is believed to foster greater responsibility and ownership of the marine ecosystem (CBD Secretariat, 2004). Though SwAM emphasizes the active participation of stakeholders as a key component in shaping the strategy, the documents do not explicitly detail how this participation occurred or how it influenced their final form. This is therefore left as an unexplored but notable aspect to our findings, worthy of further research.

Moreover, the recurring emphasis on the need for “more knowledge” in SwAM’s policy documents should not only be interpreted as a call for epistemic support, but also as a strategic feature within valuation practices themselves. As research in marine governance suggests, appeals to uncertainty or insufficient knowledge may sometimes be used to delay or steer valuation processes, effectively postponing difficult or politically sensitive decisions (Birkenholtz & Simon, 2022; Dewulf & Biesbroek, 2018; Karlsson & Gilek, 2020; Rayner, 2012). Rather than standing outside of valuation, such knowledge claims are part of it: they shape when and how values become actionable, and what forms of knowledge are mobilized to support or inhibit particular framings. This underscores how valuation and epistemic practices are not only entangled but co-produced through institutional and strategic logics. Seen this way, “knowledge gaps” are not merely obstacles to valuation but can also function as instruments of governance that determine the timing and terms of environmental action.

Yet this articulation of value occurs within a predefined ethical horizon. In SwAM’s management framework, ecological entities are rarely allowed to hold value on their own terms. Instead, fish, seals, or marine ecosystems become valuable insofar as they support human goals, whether economic, cultural, or political. This effectively marginalizes alternative ontologies and moral commitments, such as those grounded in biocentrism or reciprocity, that might challenge the prevailing economic-utilitarian framing. A shift from dominance-based to relation-based or biocentric governance would entail re-evaluating what is made valuable and rethinking the forms of knowledge environmental management relies upon. This perspective aligns with a pragmatist understanding of value, where values are not fixed or intrinsic but emerge through situated practices. It also invites reflection on how knowledge and value co-evolve through institutional practices such as those undertaken by SwAM. This poses the need for further research on the intricate interactions of valuation and knowledge production in non-anthropocentric human–nature relations.
